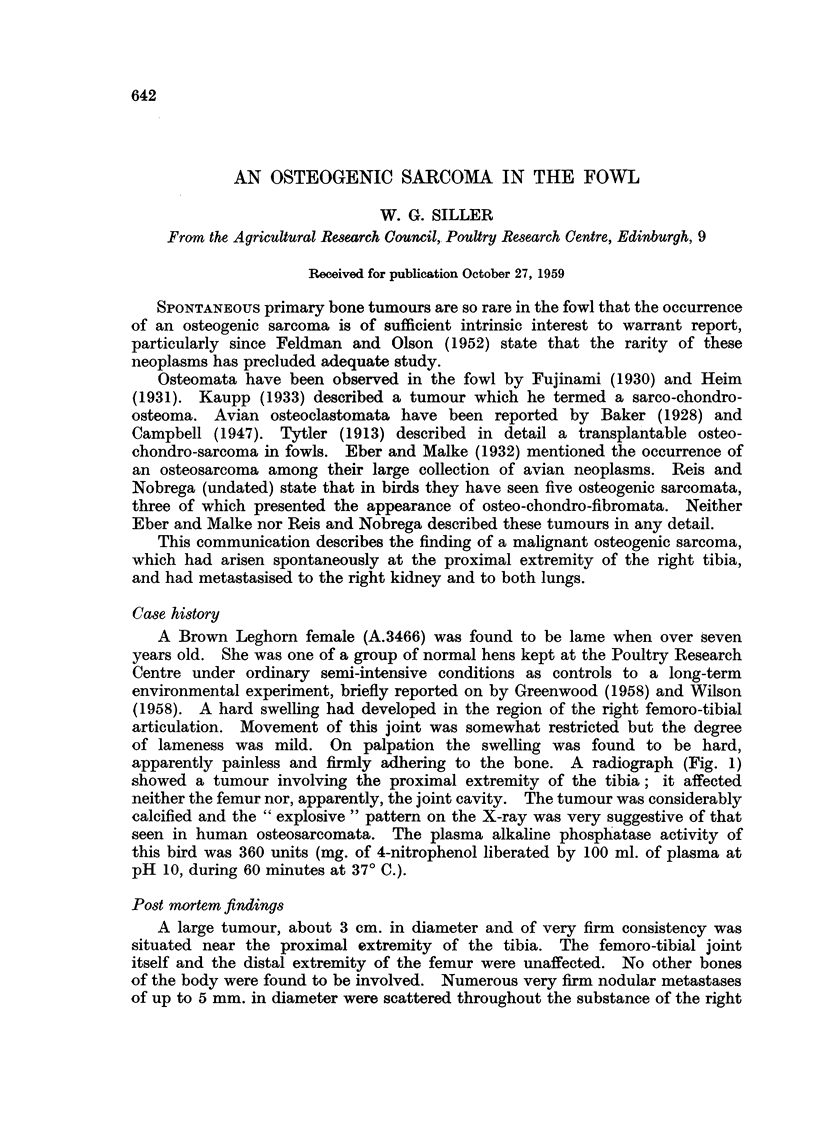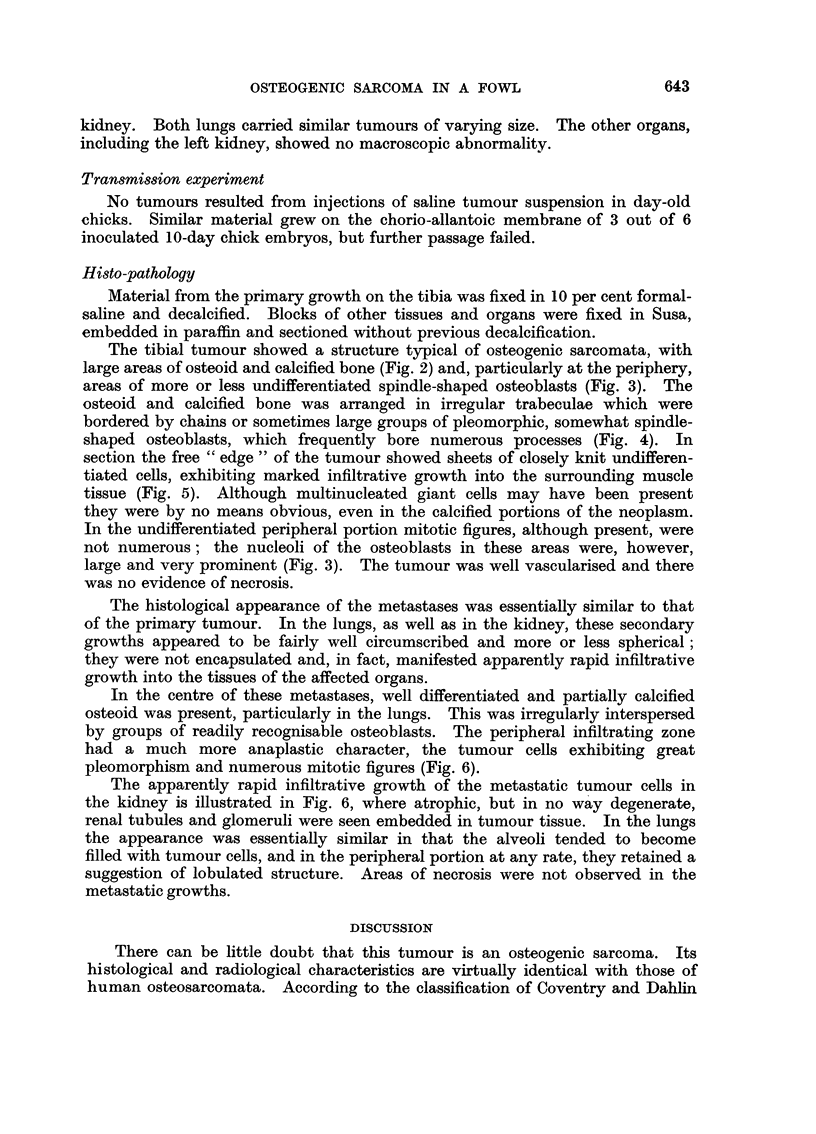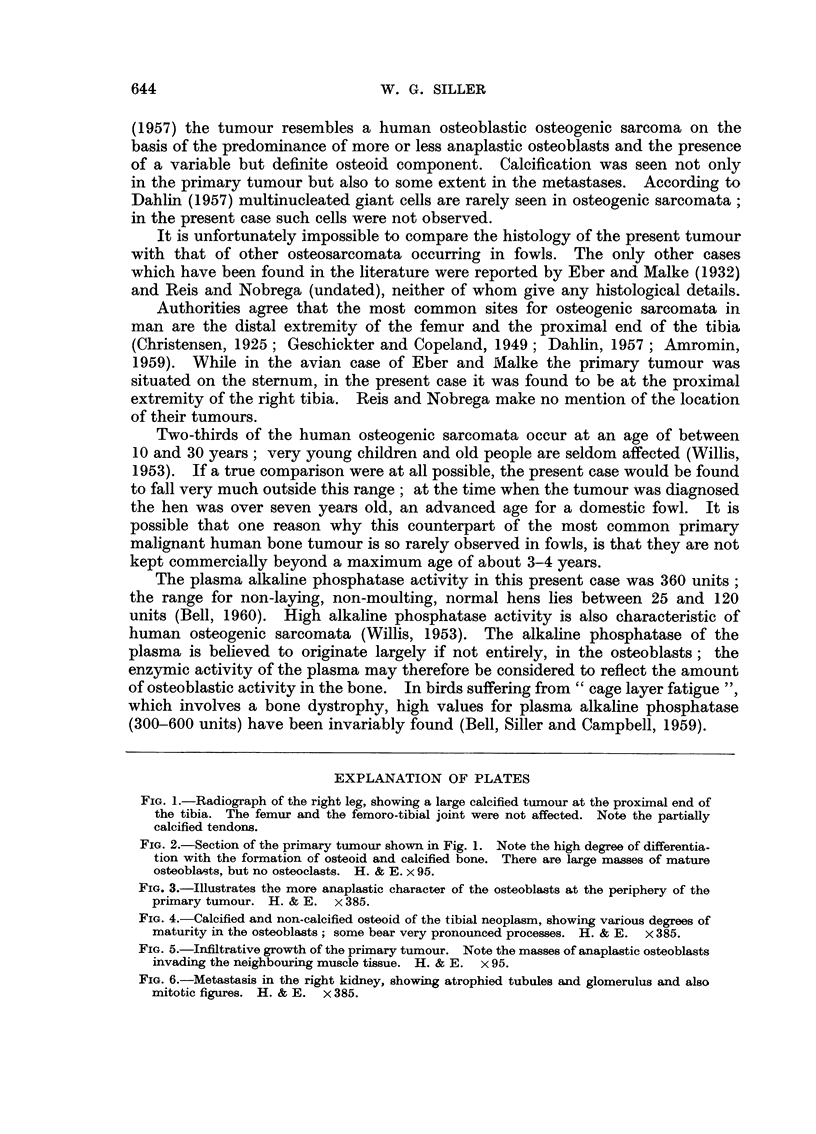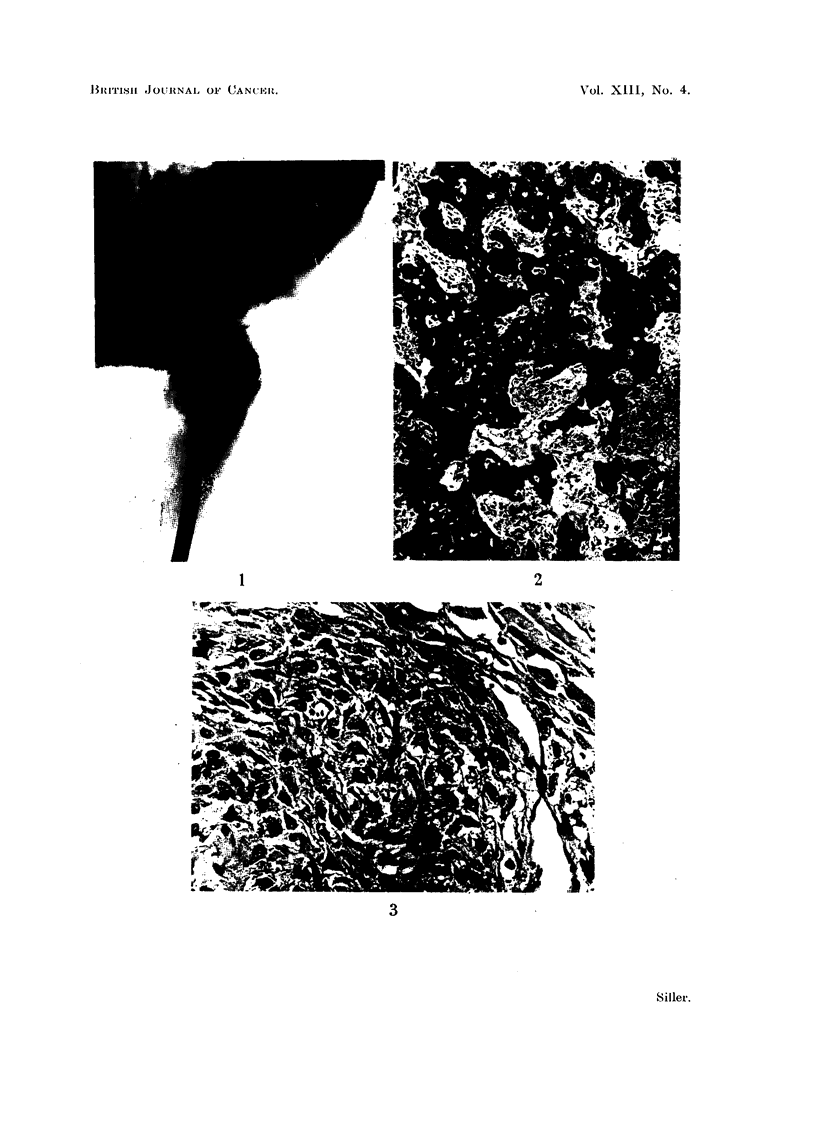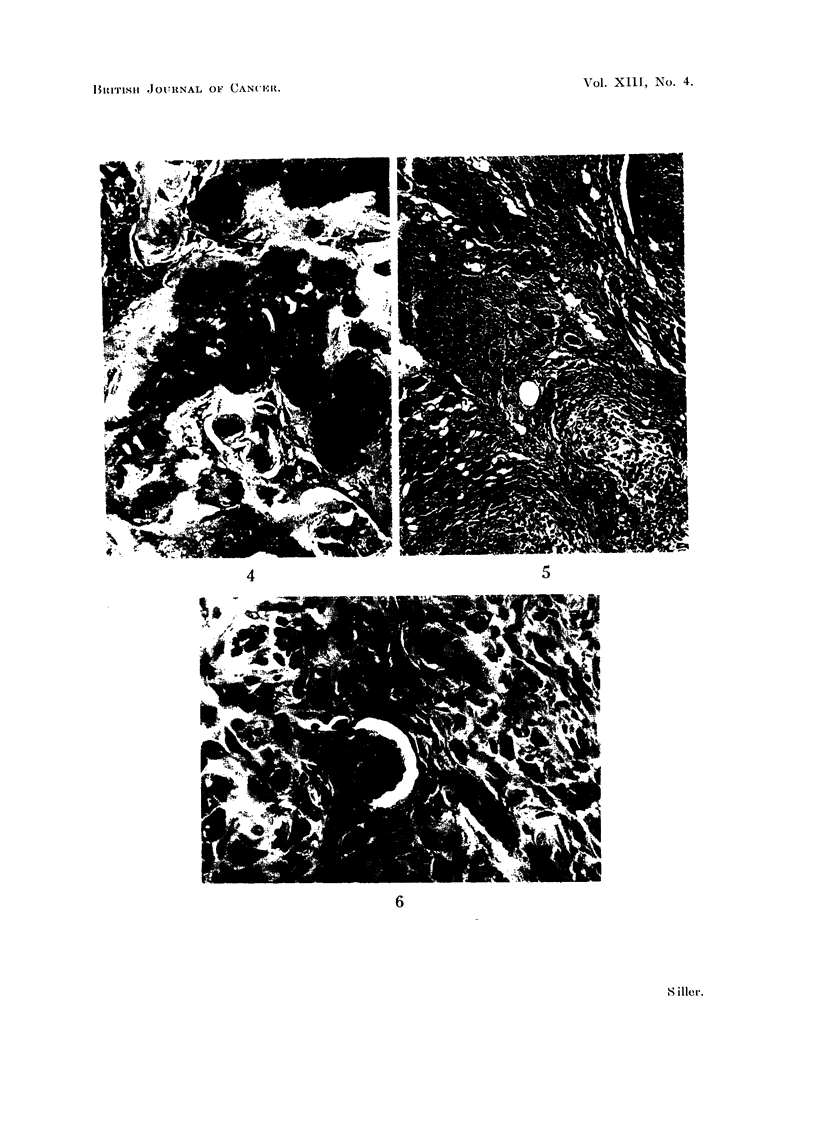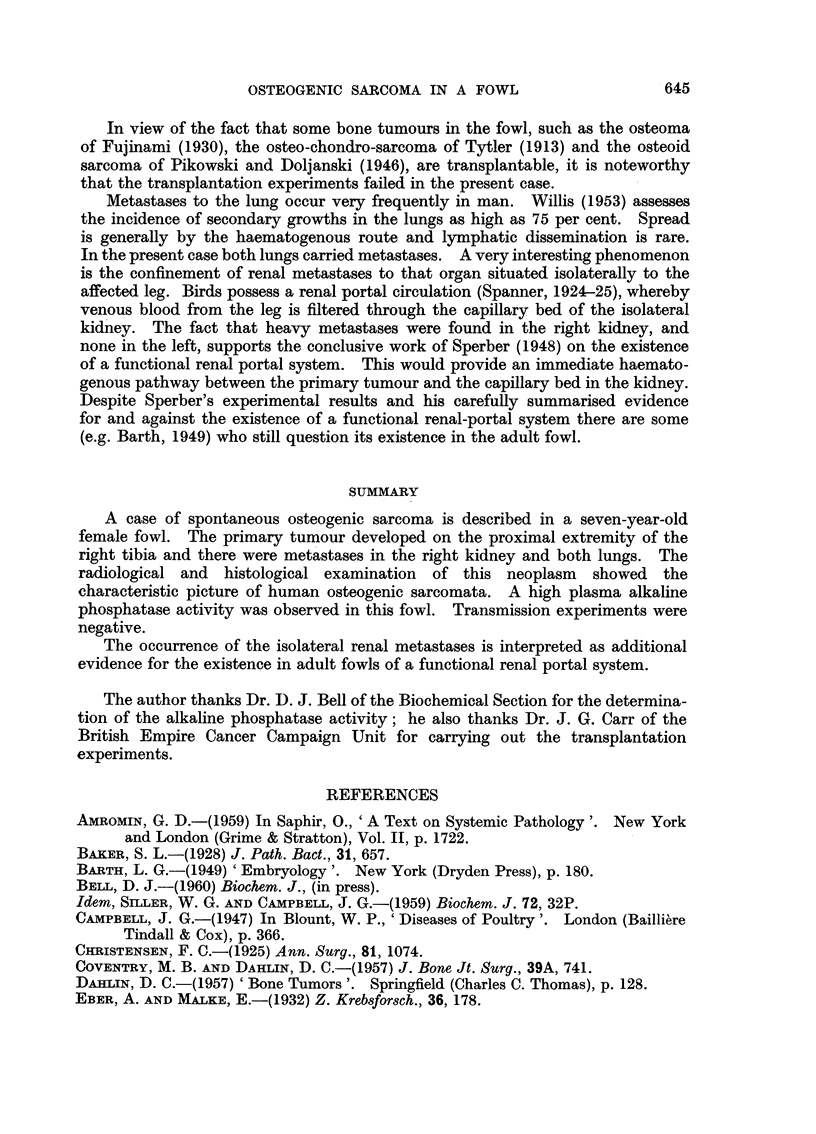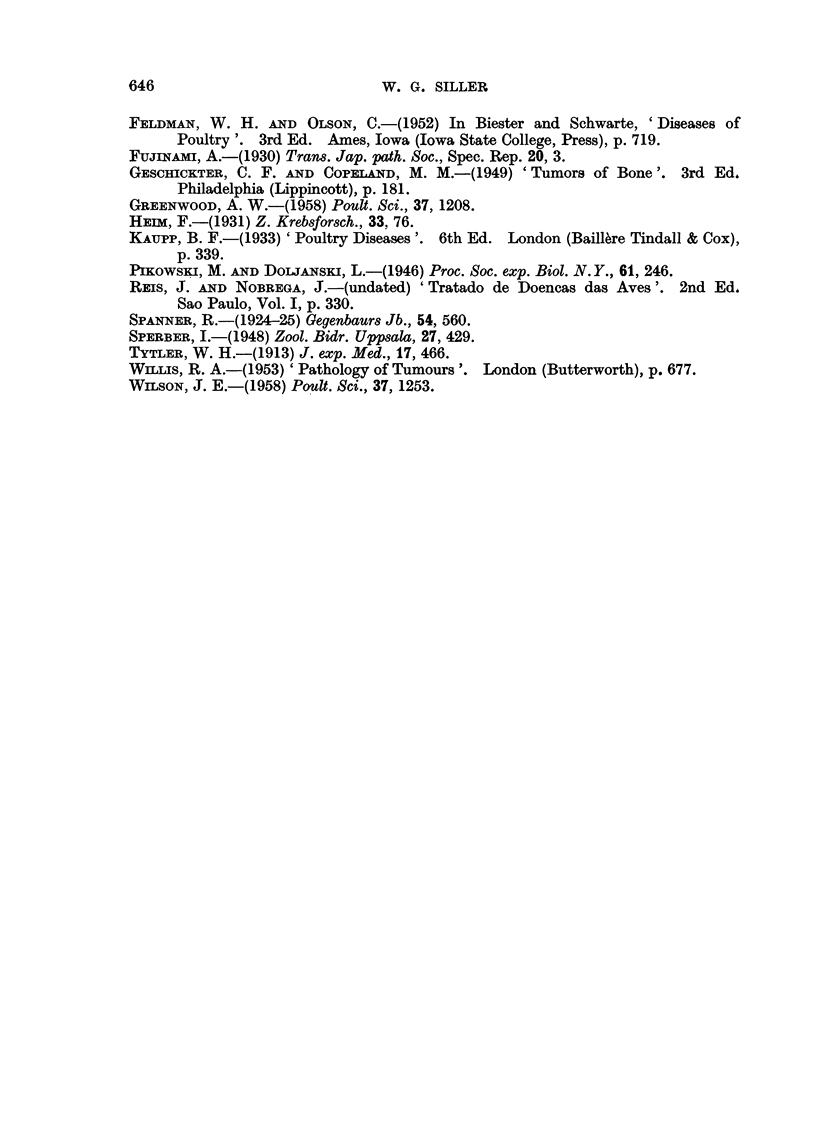# An Osteogenic Sarcoma in the Fowl

**DOI:** 10.1038/bjc.1959.70

**Published:** 1959-12

**Authors:** W. G. Siller

## Abstract

**Images:**


### AN OSTEOGENIC SARCOMA IN THE FOWL

W. G. SILLER

*From the Agricultural Research Council, Poultry Research Centre, Edinburgh, 9*

Received for publication October 27, 1959

SPONTANEOUS primary bone tumours are so rare in the fowl that the occurrence of an osteogenic sarcoma is of sufficient intrinsic interest to warrant report, particularly since Feldman and Olson (1952) state that the rarity of these neoplasms has precluded adequate study.

Osteomata have been observed in the fowl by Fujinami (1930) and Heim (1931). Kaupp (1933) described a tumour which he termed a sarco-chondro-osteoma. Avian osteoclastomata have been reported by Baker (1928) and Campbell (1947). Tytler (1913) described in detail a transplantable osteo-chondro-sarcoma in fowls. Eber and Malke (1932) mentioned the occurrence of an osteosarcoma among their large collection of avian neoplasms. Reis and Nobrega (undated) state that in birds they have seen five osteogenic sarcomata, three of which presented the appearance of osteo-chondro-fibromata. Neither Eber and Malke nor Reis and Nobrega described these tumours in any detail.

This communication describes the finding of a malignant osteogenic sarcoma, which had arisen spontaneously at the proximal extremity of the right tibia, and had metastasised to the right kidney and to both lungs.

### *Case history*

A Brown Leghorn female (A.3466) was found to be lame when over seven years old. She was one of a group of normal hens kept at the Poultry Research Centre under ordinary semi-intensive conditions as controls to a long-term environmental experiment, briefly reported on by Greenwood (1958) and Wilson (1958). A hard swelling had developed in the region of the right femoro-tibial articulation. Movement of this joint was somewhat restricted but the degree of lameness was mild. On palpation the swelling was found to be hard, apparently painless and firmly adhering to the bone. A radiograph (Fig. 1) showed a tumour involving the proximal extremity of the tibia; it affected neither the femur nor, apparently, the joint cavity. The tumour was considerably calcified and the " explosive " pattern on the X-ray was very suggestive of that seen in human osteosarcomata. The plasma alkaline phosphatase activity of this bird was 360 units (mg. of 4-nitrophenol liberated by 100 ml. of plasma at pH 10, during 60 minutes at 37° C.).

### *Post mortem findings*

A large tumour, about 3 cm. in diameter and of very firm consistency was situated near the proximal extremity of the tibia. The femoro-tibial joint itself and the distal extremity of the femur were unaffected. No other bones of the body were found to be involved. Numerous very firm nodular metastases of up to 5 mm. in diameter were scattered throughout the substance of the right kidney. Both lungs carried similar tumours of varying size. The other organs, including the left kidney, showed no macroscopic abnormality.

*Transmission experiment*

No tumours resulted from injections of saline tumour suspension in day-old chicks. Similar material grew on the chorio-allantoic membrane of 3 out of 6 inoculated 10-day chick embryos, but further passage failed.

*Histo-pathology*

Material from the primary growth on the tibia was fixed in 10 per cent formal-saline and decalcified. Blocks of other tissues and organs were fixed in Susa, embedded in paraffin and sectioned without previous decalcification.

The tibial tumour showed a structure typical of osteogenic sarcomata, with large areas of osteoid and calcified bone (Fig. 2) and, particularly at the periphery, areas of more or less undifferentiated spindle-shaped osteoblasts (Fig. 3). The osteoid and calcified bone was arranged in irregular trabeculae which were bordered by chains or sometimes large groups of pleomorphic, somewhat spindle-shaped osteoblasts, which frequently bore numerous processes (Fig. 4). In section the free " edge " of the tumour showed sheets of closely knit undifferentiated cells, exhibiting marked infiltrative growth into the surrounding muscle tissue (Fig. 5). Although multinucleated giant cells may have been present they were by no means obvious, even in the calcified portions of the neoplasm. In the undifferentiated peripheral portion mitotic figures, although present, were not numerous ; the nucleoli of the osteoblasts in these areas were, however, large and very prominent (Fig. 3). The tumour was well vascularised and there was no evidence of necrosis.

The histological appearance of the metastases was essentially similar to that of the primary tumour. In the lungs, as well as in the kidney, these secondary growths appeared to be fairly well circumscribed and more or less spherical ; they were not encapsulated and, in fact, manifested apparently rapid infiltrative growth into the tissues of the affected organs.

In the centre of these metastases, well differentiated and partially calcified osteoid was present, particularly in the lungs. This was irregularly interspersed by groups of readily recognisable osteoblasts. The peripheral infiltrating zone had a much more anaplastic character, the tumour cells exhibiting great pleomorphism and numerous mitotic figures (Fig. 6).

The apparently rapid infiltrative growth of the metastatic tumour cells in the kidney is illustrated in Fig. 6, where atrophic, but in no way degenerate, renal tubules and glomeruli were seen embedded in tumour tissue. In the lungs the appearance was essentially similar in that the alveoli tended to become filled with tumour cells, and in the peripheral portion at any rate, they retained a suggestion of lobulated structure. Areas of necrosis were not observed in the metastatic growths.

### DISCUSSION

There can be little doubt that this tumour is an osteogenic sarcoma. Its histological and radiological characteristics are virtually identical with those of human osteosarcomata. According to the classification of Coventry and Dahlin

(1957) the tumour resembles a human osteoblastic osteogenic sarcoma on the basis of the predominance of more or less anaplastic osteoblasts and the presence of a variable but definite osteoid component. Calcification was seen not only in the primary tumour but also to some extent in the metastases. According to Dahlin (1957) multinucleated giant cells are rarely seen in osteogenic sarcomata ; in the present case such cells were not observed.

It is unfortunately impossible to compare the histology of the present tumour with that of other osteosarcomata occurring in fowls. The only other cases which have been found in the literature were reported by Eber and Malke (1932) and Reis and Nobrega (undated), neither of whom give any histological details.

Authorities agree that the most common sites for osteogenic sarcomata in man are the distal extremity of the femur and the proximal end of the tibia (Christensen, 1925 ; Geschickter and Copeland, 1949 ; Dahlin, 1957 ; Amromin, 1959). While in the avian case of Eber and Malke the primary tumour was situated on the sternum, in the present case it was found to be at the proximal extremity of the right tibia. Reis and Nobrega make no mention of the location of their tumours.

Two-thirds of the human osteogenic sarcomata occur at an age of between 10 and 30 years ; very young children and old people are seldom affected (Willis, 1953). If a true comparison were at all possible, the present case would be found to fall very much outside this range ; at the time when the tumour was diagnosed the hen was over seven years old, an advanced age for a domestic fowl. It is possible that one reason why this counterpart of the most common primary malignant human bone tumour is so rarely observed in fowls, is that they are not kept commercially beyond a maximum age of about 3–4 years.

The plasma alkaline phosphatase activity in this present case was 360 units ; the range for non-laying, non-moulting, normal hens lies between 25 and 120 units (Bell, 1960). High alkaline phosphatase activity is also characteristic of human osteogenic sarcomata (Willis, 1953). The alkaline phosphatase of the plasma is believed to originate largely if not entirely, in the osteoblasts ; the enzymic activity of the plasma may therefore be considered to reflect the amount of osteoblastic activity in the bone. In birds suffering from “ cage layer fatigue ”, which involves a bone dystrophy, high values for plasma alkaline phosphatase (300–600 units) have been invariably found (Bell, Siller and Campbell, 1959).

---

### EXPLANATION OF PLATES

FIG. 1.—Radiograph of the right leg, showing a large calcified tumour at the proximal end of the tibia. The femur and the femoro-tibial joint were not affected. Note the partially calcified tendons.

FIG. 2.—Section of the primary tumour shown in Fig. 1. Note the high degree of differentiation with the formation of osteoid and calcified bone. There are large masses of mature osteoblasts, but no osteoclasts. H. & E. ×95.

FIG. 3.—Illustrates the more anaplastic character of the osteoblasts at the periphery of the primary tumour. H. & E. ×385.

FIG. 4.—Calcified and non-calcified osteoid of the tibial neoplasm, showing various degrees of maturity in the osteoblasts ; some bear very pronounced processes. H. & E. ×385.

FIG. 5.—Infiltrative growth of the primary tumour. Note the masses of anaplastic osteoblasts invading the neighbouring muscle tissue. H. & E. ×95.

FIG. 6.—Metastasis in the right kidney, showing atrophied tubules and glomerulus and also mitotic figures. H. & E. ×385.

1

2

3

Siller.

4

5

6

Siller.

In view of the fact that some bone tumours in the fowl, such as the osteoma of Fujinami (1930), the osteo-chondro-sarcoma of Tytler (1913) and the osteoid sarcoma of Pikowski and Doljanski (1946), are transplantable, it is noteworthy that the transplantation experiments failed in the present case.

Metastases to the lung occur very frequently in man. Willis (1953) assesses the incidence of secondary growths in the lungs as high as 75 per cent. Spread is generally by the haematogenous route and lymphatic dissemination is rare. In the present case both lungs carried metastases. A very interesting phenomenon is the confinement of renal metastases to that organ situated isolaterally to the affected leg. Birds possess a renal portal circulation (Spanner, 1924–25), whereby venous blood from the leg is filtered through the capillary bed of the isolateral kidney. The fact that heavy metastases were found in the right kidney, and none in the left, supports the conclusive work of Sperber (1948) on the existence of a functional renal portal system. This would provide an immediate haematogenous pathway between the primary tumour and the capillary bed in the kidney. Despite Sperber's experimental results and his carefully summarised evidence for and against the existence of a functional renal-portal system there are some (e.g. Barth, 1949) who still question its existence in the adult fowl.

### SUMMARY

A case of spontaneous osteogenic sarcoma is described in a seven-year-old female fowl. The primary tumour developed on the proximal extremity of the right tibia and there were metastases in the right kidney and both lungs. The radiological and histological examination of this neoplasm showed the characteristic picture of human osteogenic sarcomata. A high plasma alkaline phosphatase activity was observed in this fowl. Transmission experiments were negative.

The occurrence of the isolateral renal metastases is interpreted as additional evidence for the existence in adult fowls of a functional renal portal system.

The author thanks Dr. D. J. Bell of the Biochemical Section for the determination of the alkaline phosphatase activity; he also thanks Dr. J. G. Carr of the British Empire Cancer Campaign Unit for carrying out the transplantation experiments.

### REFERENCES

AMROMIN, G. D.—(1959) In Saphir, O., 'A Text on Systemic Pathology'. New York and London (Grime & Stratton), Vol. II, p. 1722.

BAKER, S. L.—(1928) *J. Path. Bact.*, **31**, 657.

BARTH, L. G.—(1949) 'Embryology'. New York (Dryden Press), p. 180.

BELL, D. J.—(1960) *Biochem. J.*, (in press).

*Idem*, SILLER, W. G. AND CAMPBELL, J. G.—(1959) *Biochem. J.* **72**, 32P.

CAMPBELL, J. G.—(1947) In Blount, W. P., 'Diseases of Poultry'. London (Baillière Tindall & Cox), p. 366.

CHRISTENSEN, F. C.—(1925) *Ann. Surg.*, **81**, 1074.

COVENTRY, M. B. AND DAHLIN, D. C.—(1957) *J. Bone Jt. Surg.*, **39**A, 741.

DAHLIN, D. C.—(1957) 'Bone Tumors'. Springfield (Charles C. Thomas), p. 128.

EBER, A. AND MALKE, E.—(1932) *Z. Krebsforsch.*, **36**, 178.

FELDMAN, W. H. AND OLSON, C.—(1952) In Biester and Schwarte, 'Diseases of Poultry'. 3rd Ed. Ames, Iowa (Iowa State College, Press), p. 719.
FUJINAMI, A.—(1930) *Trans. Jap. path. Soc.*, Spec. Rep. **20**, 3.
GESCHICKTER, C. F. AND COPELAND, M. M.—(1949) 'Tumors of Bone'. 3rd Ed. Philadelphia (Lippincott), p. 181.
GREENWOOD, A. W.—(1958) *Poult. Sci.*, **37**, 1208.
HEIM, F.—(1931) *Z. Krebsforsch.*, **33**, 76.
KAUPP, B. F.—(1933) 'Poultry Diseases'. 6th Ed. London (Baillère Tindall & Cox), p. 339.
PIKOWSKI, M. AND DOLJANSKI, L.—(1946) *Proc. Soc. exp. Biol. N.Y.*, **61**, 246.
REIS, J. AND NOBREGA, J.—(undated) 'Tratado de Doencas das Aves'. 2nd Ed. Sao Paulo, Vol. I, p. 330.
SPANNER, R.—(1924–25) *Gegenbaurs Jb.*, **54**, 560.
SPERBER, I.—(1948) *Zool. Bidr. Uppsala*, **27**, 429.
TYTLER, W. H.—(1913) *J. exp. Med.*, **17**, 466.
WILLIS, R. A.—(1953) 'Pathology of Tumours'. London (Butterworth), p. 677.
WILSON, J. E.—(1958) *Poult. Sci.*, **37**, 1253.